# M-BISON: Microarray-based integration of data sources using networks

**DOI:** 10.1186/1471-2105-9-214

**Published:** 2008-04-25

**Authors:** Bernie J Daigle, Russ B Altman

**Affiliations:** 1 Department of Genetics, Stanford University School of Medicine, Stanford, CA 94305, USA; 2 Department of Bioengineering, Stanford University School of Engineering, Stanford, CA 94305, USA

## Abstract

**Background:**

The accurate detection of differentially expressed (DE) genes has become a central task in microarray analysis. Unfortunately, the noise level and experimental variability of microarrays can be limiting. While a number of existing methods partially overcome these limitations by incorporating biological knowledge in the form of gene groups, these methods sacrifice gene-level resolution. This loss of precision can be inappropriate, especially if the desired output is a ranked list of individual genes. To address this shortcoming, we developed M-BISON (Microarray-Based Integration of data SOurces using Networks), a formal probabilistic model that integrates background biological knowledge with microarray data to predict individual DE genes.

**Results:**

M-BISON improves signal detection on a range of simulated data, particularly when using very noisy microarray data. We also applied the method to the task of predicting heat shock-related differentially expressed genes in *S. cerevisiae*, using an *hsf1 *mutant microarray dataset and conserved yeast DNA sequence motifs. Our results demonstrate that M-BISON improves the analysis quality and makes predictions that are easy to interpret in concert with incorporated knowledge. Specifically, M-BISON increases the AUC of DE gene prediction from .541 to .623 when compared to a method using only microarray data, and M-BISON outperforms a related method, GeneRank. Furthermore, by analyzing M-BISON predictions in the context of the background knowledge, we identified YHR124W as a potentially novel player in the yeast heat shock response.

**Conclusion:**

This work provides a solid foundation for the principled integration of imperfect biological knowledge with gene expression data and other high-throughput data sources.

## Background

By measuring the abundance of tens of thousands of mRNA transcripts at once, DNA microarrays have become an important laboratory tool for analyzing gene expression and its regulation over a wide range of conditions and cell types. Since their introduction in 1995 [1], microarrays have measured expression in both large-scale multi-condition assays as well as individual condition queries. Though multi-condition analyses are most well known, individual condition queries are common, as most labs are interested in one or a few phenotypes and cannot afford to test all possible perturbations of a given cell type or organism. In particular, microarrays are often used to detect *differentially expressed (DE) genes *– which are defined here as genes whose transcripts are expressed at different levels between two conditions. In this work we consider differential expression to be a binary state; to estimate this state we ask whether the level of measured expression of each gene in the two conditions is significantly different.

Unfortunately, even though limiting the number of tested conditions simplifies the experimental protocols, it also creates a problem. Typically, only a small number of genes are differentially expressed between two conditions; it therefore becomes difficult to separate the biologically relevant genes from the vast majority of genes that are unchanged or whose changes are artifactual. This stems in large part from the inherent noisiness and often poor reproducibility of the microarray assay, especially with respect to genes expressed at low levels [2,3]. In addition, though differential expression (as we have defined it) is a binary characteristic, some genes undergo a greater fold change increase/decrease than others. It is relatively easy to detect differentially expressed genes with large changes in expression; the challenge is detecting those genes that exhibit a statistically significant but small fold change. Importantly, there is no guarantee that the most biologically relevant genes are those with the most extreme changes in expression; indeed, "innocent bystander" expression changes – unrelated to the phenotype of interest – may dilute the signal of the assay [4]. The difficulty in sensitively detecting expression changes is in part a result of methods that treat each transcript on the chip as an independent biological and statistical entity [5]. Examples of such methods include the t test, SAM, and the B statistic [6-8]. Yet it is widely accepted that gene products act together in networks to carry out their functions [9]. Although our knowledge of these networks is incomplete, it is attractive to have methods that use this information.

Experimental approaches to these analysis challenges include performing more biological replicates and validating results using more precise assays such as qRT-PCR or protein quantification, but the former solution has a practical limit [10] and both approaches are resource-limited. Therefore, informatics solutions offer an opportunity to leverage dependencies and functional relationships between genes to better identify DE genes. Lu, Liu et al. use a multivariate statistic to test for DE genes, though the dependency information here comes only from the microarray data and ignores additional known biology [5]. More commonly, researchers use methods that incorporate prior biological knowledge into microarray analysis to enhance the experimental signal. The knowledge can come from other high-throughput genomic assays, like chromatin immunoprecipitation or protein-protein interaction assays, or more commonly, from gene functional annotation, like Gene Ontology terms [11] or KEGG pathway information [12]. Of these methods, nearly all sacrifice resolution at the DE gene level in favor of aggregated statistics associated with the presumably more robust DE "gene group" level. Thus, several statistical approaches seek enrichment of gene groups in the high or low scoring end of a ranked list derived from a single condition microarray [13-18]. The latter three methods have the advantage of not requiring an arbitrary cut-off of significant vs. non-significant genes, and all methods have been used with some success in detecting DE gene groups. However, there are certainly situations where gene-level resolution is more useful than gene group resolution, such as a search for candidate genes or potential biomarkers. While the members of the highest scoring gene groups can be a source of high scoring genes, it is not clear how to consistently compare the significance of genes from different groups. Additionally, certain forms of biological knowledge, such as binary protein-protein interaction data, are not easily amenable to methods requiring discrete gene groupings.

Thus, we need a formal, consistent method to incorporate known biological relationships with gene expression data to identify differential expression at gene-level resolution. Such an approach should yield more accurate and reproducible identification of DE genes than would be possible with any data source alone or with a less sophisticated data integration strategy. One such method, GeneRank [19], uses the same principles as Google's PageRank algorithm [20] to rank differentially expressed genes based on gene expression data and prior biological knowledge. This method relies on a parameter *d*, which governs the relative weight given to knowledge versus expression data. The authors of GeneRank suggest setting *d *to 0.5 for general use, but it is clear even in simulated studies that the optimal choice for this parameter varies by dataset and there is no obvious way to choose such a value. In addition, the method validation used simulations and perturbations of existing data; thus it is not known how well the approach would work in a traditional unsupervised learning task with a proper gold standard.

In this paper, we develop, implement, validate, and apply to an experimental dataset M-BISON (Microarray-Based Integration of data SOurces using Networks), a formal probabilistic model that integrates biological knowledge with microarray data to better identify DE genes. Our model lets us assess the significance of each gene's difference in measured expression level between the conditions of interest, accounting for experimental noise and expected correlations in expression with other genes based on biological knowledge. M-BISON relies on two parameters, *α*^(*DE*) ^and *α*^(*NDE*)^, and we introduce a strategy to choose these parameter values. We use a simulation study to establish the limits of M-BISON's ability to improve results, both in terms of microarray data accuracy and knowledge structure. We then apply the method to a real world biological dataset by predicting heat shock-related DE genes from a *Saccharomyces cerevisiae hsf1 *mutant microarray dataset [21], conserved yeast DNA sequence motifs [22], and a gold standard based on Hsf1p chromatin immunoprecipitation assays [23]. In so doing, we assess the accuracy and interpretability of M-BISON's predictions, and we compare M-BISON's performance to that of GeneRank.

## Results

### Simulated data

**Figure 1:**
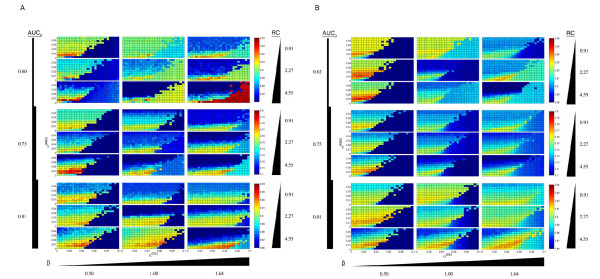
**Effects of AUC_0_, *β*, and *RC *on M-BISON performance**. Simulated datasets consist of 1000 genes, with either 10% (A) or 20% (B) DE. We measured performance using AUC of the ROC curve, plotted as a function of *α*^(*DE*) ^and *α*^(*NDE*)^. Pseudocolor represents AUC magnitude, with dark blue the lowest and dark red the highest (best performance). All simulated data runs contain at least one parameter combination that scores better than the B statistic with microarray data alone (lower left hand corner of each plot), except for AUC_0 _= 0.91/10% DE/*β *= 1.64/*RC *= 0.91.

We simulated six 1000 gene microarray datasets with either 100 or 200 DE genes (A-F) and nine knowledge sources (1–9) with a variety of configurations for a total of 54 data-knowledge combinations (Table 1). We ran M-BISON on each of these 54, using a grid of parameter values composed of all pairwise combinations of $\left\{\alpha_{i}^{\left(NDE\right)}\right\}$, ranging from 0 to .052, and $\left\{\alpha_{j}^{\left(DE\right)}\right\}$, ranging from 0 to .1, both in .004 increments (see Methods for notation details). For each run at each parameter combination ***α***_***t***_= [*α *_*i *_^(*NDE*) ^*α *_*j *_^(*DE*)^]^*T*^, we used the resultant DE scores B*(***α***_*t*_) compared to the known truth to estimate an area under the curve (AUC) of the receiver operating characteristic (ROC) curve. Figure 1 displays the results for all data-knowledge sets. With all but one of the 54 (dataset C + knowledge set 3 is the exception), M-BISON at one or more parameter combinations yields a higher AUC than what is achieved using microarray data alone in conjunction with the B statistic [24] (denoted AUC_0_).

**Table 1:** Parameters used to generate 54 simulated data combinations of 1000 genes each

Simulated microarray data		
Dataset	AUC_0_	# DE

A	0.60	100
B	0.75	100
C	0.91	100
D	0.62	200
E	0.75	200
F	0.91	200

		
Simulated biological knowledge		

Knowledge	*β*	*RC*

1	0.50	0.91
2	1.00	0.91
3	1.64	0.91
4	0.50	2.27
5	1.00	2.27
6	1.64	2.27
7	0.50	4.55
8	1.00	4.55
9	1.64	4.55

**Figure 2:**
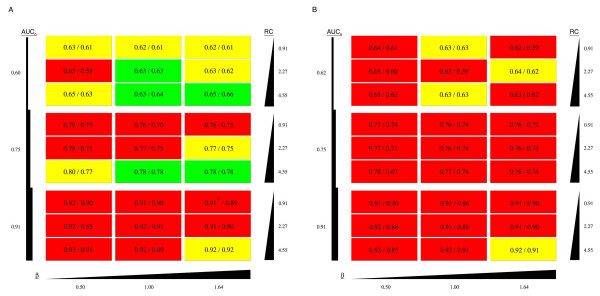
**Comparison of M-BISON and B statistic performance**. Simulated datasets are arranged as in Figure 1, one rectangle per dataset, with varying AUC_0_, *β*, *RC*, and number of DE genes. (A) 10% of genes are considered DE; (B) 20% of genes are considered DE. The first number on each dataset is the AUC for single parameter M-BISON (MB1); the second is the AUC for empirical M-BISON (MBe). Colors are used to clarify the difference in performance between using M-BISON and using the B statistic with microarray data only (MA): Green – MBe yields the highest AUC, followed by MB1 and finally MA; Yellow – MB1 yields the highest AUC, followed by MBe and finally MA; Red – MB1 yields the highest AUC, followed by MA and finally MBe. *MA AUC (AUC_0_) is slightly higher than MB1 AUC.

It is clear from Figure 1 that the optimal parameter values vary for different data-knowledge combinations. As detailed in the Methods section, standard parameter estimation approaches were not successful in yielding good performance, so we implemented an empirical approach to combine results from a large number of runs using different parameter combinations. We used this approach on each of the 54 simulated data-knowledge sets, this time on a grid where both $\left\{\alpha_{i}^{\left(NDE\right)}\right\}$ and $\left\{\alpha_{j}^{\left(DE\right)}\right\}$ ranged from 0 to .51 in .03 increments. In this case the scores $P_{\min}^{*}$ were used to estimate AUC of the ROC curves. Figure 2 shows a comparison of the performances of these scores, the B* generated using optimal parameter values, and the B statistics from microarray data alone. Using the empirical approach, M-BISON achieves higher AUCs than AUC_0 _for a defined subset of the simulated data+knowledge sets. In particular, our approach performs best when the array data are noisiest (AUC_0 _≤ .75), the number of truly DE genes is low (100/1000), and when the knowledge imparts a high relative connectivity (*RC *= 4.55) and mean degree among DE genes (*β *≥ 1). These are precisely the conditions for which M-BISON was developed.

### Yeast dataset validation

**Figure 3:**
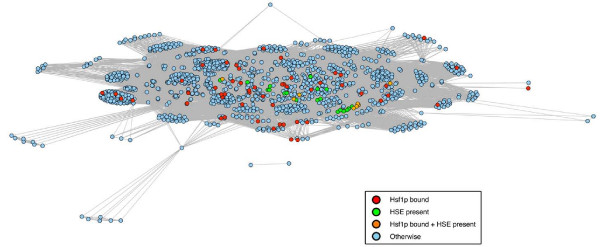
**Network representation of yeast conserved motif knowledge**. Shown are 1297/6068 genes from the *hsf1 *microarray dataset having at least one upstream motif, as determined from Kellis, Patterson et al. [22] using a score cutoff of 55. Genes are connected to each other if they share at least one motif and colored according to their Hsf1p-bound/HSE-containing status.

**Figure 4:**
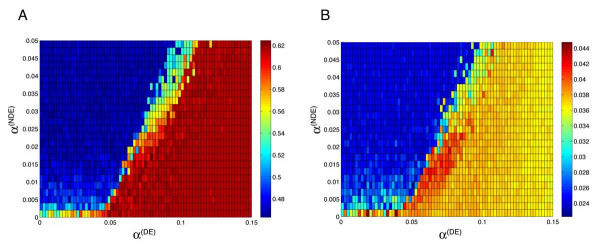
**M-BISON performance on *hsf1 *dataset as a function of *α*^(*DE*) ^and *α*^(*NDE*)^**. Dataset has the following structure: *MD *= 21.29; *RC *= 2.44; *β *= 2.05; *MCC *= 0.83. Pseudocolor represents AUC (A) and pAUC^.2 ^(B) magnitude. Ranges of high-scoring parameter values in both plots overlap to yield a "sweet spot" where parameters should be chosen for optimal performance.

We tested M-BISON on a yeast microarray dataset querying the response to a C-terminal regulatory domain mutation of the *hsf1 *transcription factor (TF) gene and knowledge consisting of genome-wide conserved intergenic motifs (Figure 3 shows a network representation of the knowledge). The domain disrupted by the *hsf1 *mutation is known to affect the transcription of Hsf1p targets [25,26], so the comparison between wild type and mutant represents an individual condition query for which M-BISON was developed. The collection of motifs is likewise relevant, because Hsf1p recognizes its targets through a conserved upstream motif [27]. We treated physical binding sites of Hsf1p as a silver standard for DE genes. Clearly direct targets of Hsf1p should be affected by the *hsf1 *mutation, although this standard will miss other genes whose regulation is downstream of the physical targets. Thus the standard will be better at confirming positive predictions than disproving them. We chose a grid of parameter values that was slightly wider than that used on the simulated data, and for each combination we calculated both standard AUC and partial AUC at a false positive rate of 0.2 (denoted pAUC^.2^). Figure 4 displays the results for both measures. M-BISON with a large range of parameter values yields better AUC/pAUC^.2 ^values than the B statistic with microarray data alone, and a subset of this range yields both maximal AUC and pAUC^.2^.

**Figure 5:**
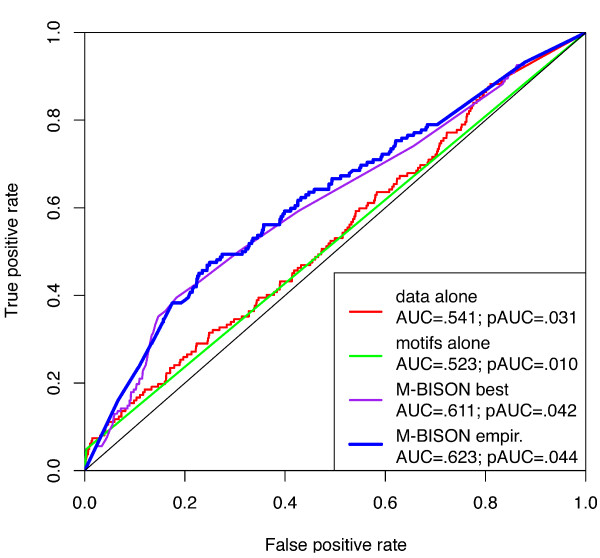
**Performance in predicting likely DE genes (Hsf1p binding) using *hsf1 *microarray data and conserved motif knowledge**. ROC curves for the B statistic with microarray data alone, motif knowledge alone, single parameter M-BISON ("best"), and empirical M-BISON ("empir.") are shown. Both forms of M-BISON significantly outperform data or knowledge alone, with the empirical method yielding the best AUC and pAUC^.2^.

Next, we tried the empirical method on the yeast data and knowledge, using the same size grid as in Figure 4. The resulting ROC curve is displayed in Figure 5, along with the curves for an optimal single parameter combination run of M-BISON ("single parameter M-BISON"), the microarray data alone with B statistic, and the knowledge alone (achieved by calling all genes that have an intergenic upstream HSE differentially expressed). Empirical M-BISON gives the best performance, at a substantial AUC improvement over microarray data or knowledge alone.

**Figure 6:**
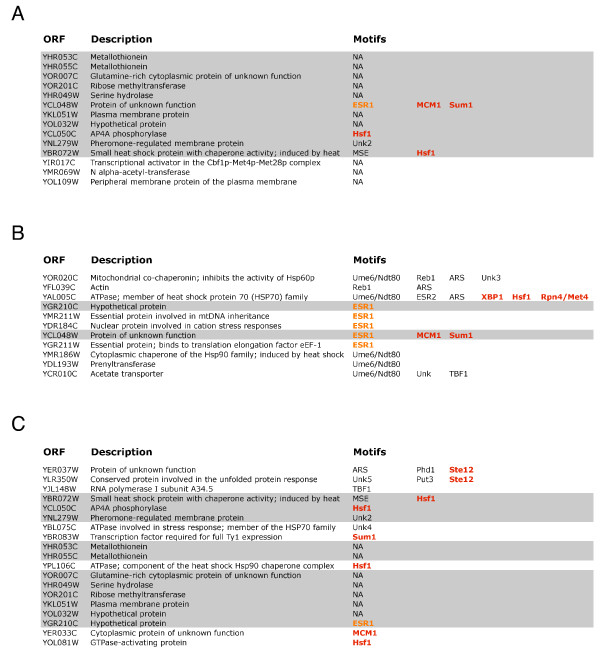
**True positive Hsf1p bound genes in the top 250 scoring genes**. Results are shown for the B statistic with microarray data only (A), M-BISON single parameter (B), and M-BISON empirical (C) approaches. Motifs in red lettering are directly related to heat shock/stress response; motifs in orange lettering are peripherally related. Genes highlighted in gray are also present in one of the other lists.

**Figure 7:**
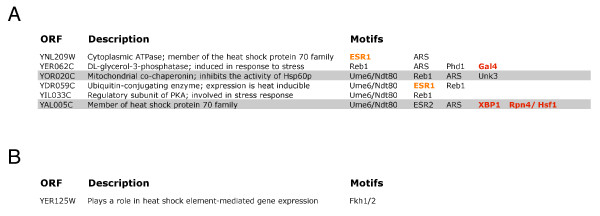
**Genes involved in stress or heat response in the top 50 scoring genes**. Results are shown for the M-BISON single parameter (A) and the B statistic (B) approaches. Coloring is the same as in Figure 6.

A closer look at the true positive genes with the highest scores reveals that using the B statistic with microarray data alone, single parameter M-BISON, or empirical M-BISON leads to somewhat different answers. Figure 6 shows lists of true positives (based on Hsf1p binding data) in the top 250 scoring genes for each of these three approaches along with their associated biological knowledge. Motifs colored orange or red are related at least peripherally to heat or stress response. We also looked in detail at the top 50 high-scoring genes of the B statistic and single parameter M-BISON for genes involved in stress or heat response. Figure 7 shows these two lists.

### Comparison to related method

**Figure 8:**
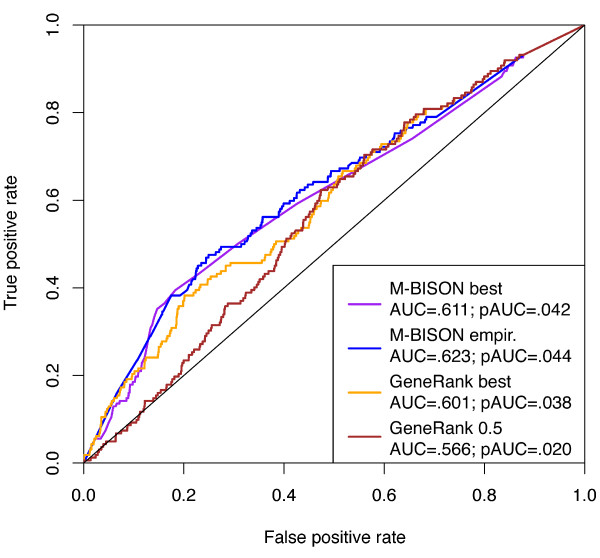
**Comparing M-BISON and GeneRank performance on the *hsf1 *dataset**. ROC curves for single parameter M-BISON, empirical M-BISON, the best performance of GeneRank (*d *= .99), and GeneRank with recommended *d *of .5 are shown. Both versions of M-BISON outperform the two instances of GeneRank. Notably, a comparison of the versions of M-BISON and GeneRank that do not require knowledge of the right answer – empirical (blue) and *d *= .5 (brown), respectively-shows that M-BISON performs significantly better in the unsupervised identification of Hsf1p bound genes.

Figure 8 shows ROC curves for the best performance of GeneRank and performance using the recommended value of *d *along with M-BISON single parameter and empirical method curves. Both versions of M-BISON outperform the two instances of GeneRank in terms of AUC and pAUC^.2^. As single parameter M-BISON and GeneRank with the best performing *d *require knowledge of the right answer, the fairest comparison is between empirical M-BISON and GeneRank with recommended *d*. In this case, M-BISON gives an AUC of .623; GeneRank achieves an AUC of .566.

### Differential expression stories

**Figure 9:**
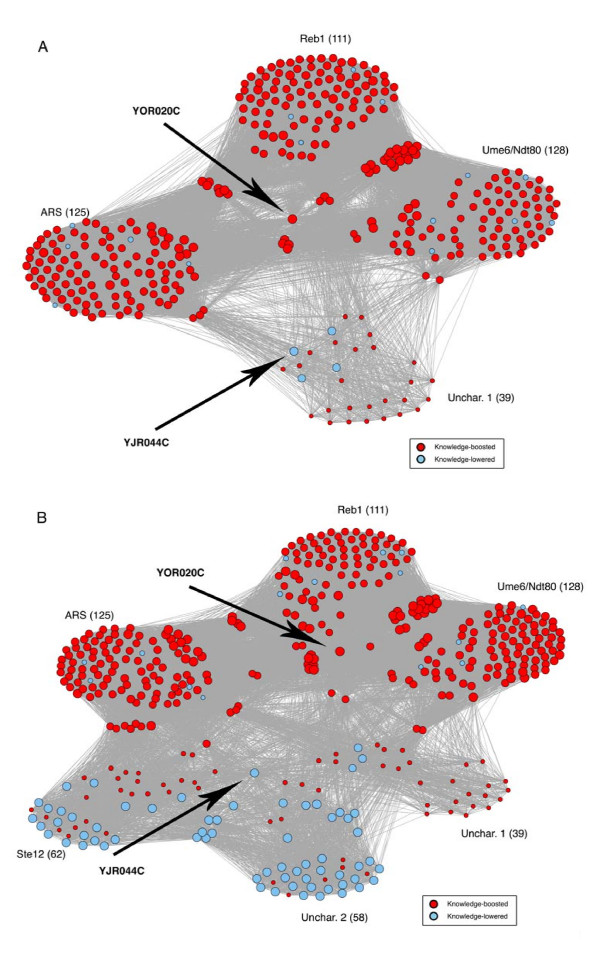
**YOR020C and YJR044C differential expression stories**. Stories are shown for YOR020C alone (A) and YOR020C + YJR044C (B) from the *hsf1 *dataset. Gene names have been removed for clarity. Black arrows mark the two genes. Large clusters of genes represent individual upstream motifs. Sizes of red (boosted with respect to original rank) genes are directly proportional to magnitudes of M-BISON scores; sizes of blue (lowered with respect to original rank) genes are inversely proportional to score magnitudes. YOR020C is boosted in score by M-BISON, and it is clear that this is due to the many boosted genes surrounding it in knowledge. In contrast, YJR044C receives a lower score due to the motifs, in spite of many boosted genes surrounding it. By expanding the DE story in (B), the reason for this is revealed: YJR044C is connected through two additional motifs to 61 genes that are also lowered.

We chose to further explore M-BISON's impact on two intriguing genes from the *hsf1 *datasets in the context of their biological knowledge. We created a "differential expression story" for each gene, which displays M-BISON's effect on the gene of interest and all knowledge-associated genes, along with all of their connections. Figure 9a shows the DE story of YOR020C (*hsp10*), an Hsf1p-bound gene and the highest-scoring true positive hit for single parameter M-BISON. Encoding a mitochondrial chaperonin, YOR020C's DE score ranks 2762 out of 6277 based on the B statistic with microarray data alone. Upon application of M-BISON, the score is boosted to a rank of 23. YOR020C contains four upstream motifs: Reb1, Ume6/Ndt80, ARS, and an uncharacterized conserved sequence. It is worth noting that the absence of an HSE preceding this gene suggests, as is supported in Figure 5, that the HSE is not necessary for the physical binding of Hsf1p. The vast majority of the 361 genes in YOR020C's DE story have similarly high DE scores, explaining the large boost the gene receives from M-BISON. However, four genes in the story: YJR044C, YMR182C, YMR145C, and YOR310C earn relatively low M-BISON DE scores. An expansion of the story in Figure 9b to include all of YJR044C's knowledge neighbors illustrates why this gene receives a low score: two additional motifs (Ste12 and uncharacterized) are present upstream of YJR044C that connect it to 61 low-scoring genes.

**Figure 10:**
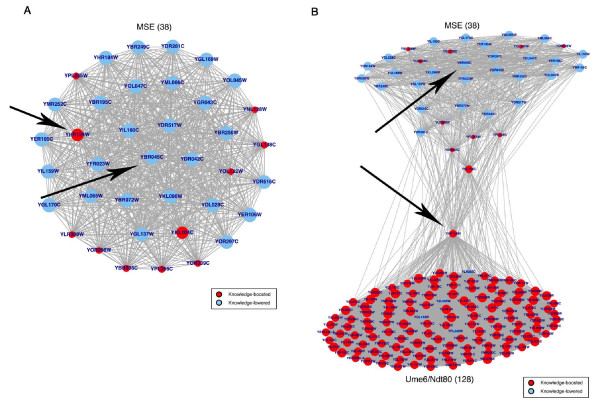
**YBR045C and YHR124W differential expression stories**. Stories are shown for YBR045C alone (A) and YBR045C + YHR124W (B). Sizing and coloring conventions are the same as in Figure 9. YBR045C is lowered in score by M-BISON due to the many lowered genes sharing the MSE motif. YHR124W is boosted in spite of these genes, and the expansion in (B) shows that this is due to the many boosted genes that also contain the Ume6/Ndt80 motif.

Figure 10a displays the DE story of YBR045C (*gip1*), whose gene product is a regulatory subunit for a meiotic phosphatase. This gene has no known relationship to Hsf1p or heat shock/stress response, and an original DE score rank of 229 is lowered to 5144 by single parameter M-BISON. YBR045C contains one upstream motif, MSE, which another 37 genes share. The majority of these genes also earn low M-BISON scores, supporting the lowering of YBR045C's score. Surprisingly, two genes in the story, YHR124W and YKL104C, receive high M-BISON DE scores. By including all of YHR124W's (*ndt80's*) neighbors in the DE story (Figure 10b), we see that the reason it received a boost is the Ume6/Ndt80 motif present upstream of it and another 126 genes, most of which also have high scores. YHR124W itself is boosted from an original rank of 4596 to 365 because of this motif. While this TF has no known association with heat shock or stress response, this score boost coupled with the fact that its own motif is found upstream of 5 of the 11 correctly called Hsf1p-bound genes (Figure 5) suggests that its role in these pathways might warrant further investigation. To more rigorously confirm this notion, we assayed whether any of the 27 motifs with an unambiguously defined TF were enriched in the top 5% M-BISON scoring genes (single parameter list). Of these, we filtered out those motifs whose TF gene did not have an M-BISON score within the top 10% of genes. Two TFs satisfied these criteria and were statistically significant according to the hypergeometric p-value: YKL112W (p < 1.2e-06), a DNA repair TF, and YHR124W (p < 4.5e-83).

## Discussion

A robust and properly validated method to incorporate known biological relationships with gene expression data to identify gene-wise differential expression would be useful and has not been available. To address this need, we created M-BISON, a probabilistic approach to integrating biological knowledge with microarray data.

One might argue that in microarray expression analyses, we should "let the data speak for themselves" and not bias the analysis of differential expression with potentially inaccurate or irrelevant biological knowledge. There are a few points to make. First, it is always possible to run unbiased analyses given the experimental data (this of course should be done), but the data may be speaking in a noisy room with echoes, in which case having some idea of what the data may be saying could assist in interpretation. Second, our method (as illustrated in the case studies of YOR020C and YBR045C) is an effective way to formally define "surprising results" and thus focus attention on potentially novel findings. If the analysis with biological knowledge and the unbiased analysis lead to marked changes in rank for a gene (YOR020C moved from 2762 in the unbiased analysis to 23 with M-BISON) then it deserves study; there is clearly an interesting tension between the data and current knowledge worth examining. If the biased and unbiased analyses rank a gene similarly, then the data and knowledge roughly agree and so a startling discovery is less likely. Thus, M-BISON allows correct knowledge to assist the analysis and highlights inconsistencies so that potentially incorrect knowledge can be identified and reconsidered.

Biological knowledge can take many forms, including gene functional annotation, protein-protein interactions, DNA motifs, and microarray data compendia. Each of these sources can readily be represented as a graph whose nodes are genes and whose edges connect genes that either share a biological function, code for interacting protein products, contain the same upstream DNA motif, or correlate significantly across array experiments. Using these graphs, we are able to represent knowledge ranging from labelled groups (e.g. Gene Ontology terms) to binary connections (e.g. protein interaction data) in a format amenable to a graphical model. In this manner, M-BISON can incorporate many different kinds of biological knowledge and treat them identically, governing their degree of influence by two parameters *α*^(*NDE*) ^and *α*^(*DE*)^. Intuitively, *α*^(*NDE*) ^and *α*^(*DE*) ^determine the amount of weight we give to the knowledge to decrease and increase, respectively, the overall DE score for each gene. With these two parameters set to zero, the scores are calculated from the microarray data only; as the parameters' values increase, knowledge plays a larger and larger role.

Using simulated data, we tested M-BISON on a variety of data/knowledge structures and, using an empirical approach for combining results from runs obtained with different parameter values, demonstrated better performance in identifying DE genes in a subset of these structures. We acknowledge that such an empirical approach is unconventional in the context of undirected probabilistic models, but as mentioned in the Methods section, standard parameter estimation strategies were ineffective. Specifically, empirical M-BISON works well when applied to noisy microarray data (low AUC_0_), conditions resulting in a small number of DE genes, and knowledge sources with a high relative connectivity and/or high *β *value. While these characteristics might be viewed as limitations, we note they are also precisely the conditions that call for a method like M-BISON. First, improving DE gene identification with expression data is only really necessary when the data are noisy. Second, as the cost of additional experimentation often limits how many DE genes can be pursued in the lab, a method giving higher quality DE predictions is more useful when the overall number of DE genes is expected to be low. If there are many DE genes, experiments can be restricted to the most significant DE gene candidates, which even when identified with a method using only microarray data are likely to be correct. Finally, a knowledge source that is relevant to the conditions of the microarray experiment will likely have a high relative connectivity and *β *value. We note that while it is impossible to know the exact values of the above features without knowing the right answer, rough estimates of the first two can be obtained from an analysis of the microarray data, while the third can be made favorable by careful choice of the knowledge source.

The above three conditions pertain only to using empirical M-BISON. In our simulation studies, we have shown that using M-BISON with optimal parameter values yields better DE gene identification over a wide range of data and knowledge structures. Though the optimal parameter values vary for differing data-knowledge configurations, they are generally the same for different instances of the same configuration (data not shown). Thus, knowing the (in general, unknown) configuration of the data and biological knowledge would allow intelligent choosing of parameter values and expand the number of examples where M-BISON would be useful. We are currently focusing on this task.

We validated M-BISON on a yeast microarray dataset by showing improved performance in heat shock-related DE gene prediction. We used genome-wide conserved motif data as knowledge and Hsf1p transcription factor binding data as an approximation to a DE gene gold standard (silver standard). Using empirical M-BISON, we show a substantial increase in AUC and pAUC^.2 ^when compared to performance using the B statistic with array data alone. Figure 6 illustrates the differences in high-scoring true positives between the B statistic, single parameter M-BISON, and empirical M-BISON. The empirical method gives the longest list, though the overlap with the B statistic is extensive. The genes not present in the B statistic list are those that became significant due to their associated knowledge; there are nine such genes in the top 250 when using the empirical approach. Most of these genes make good biological sense: the top two genes YER037W and YLR350W share an Ste12 motif, predicted to be involved in stress response [28], and YBL075C and YPL106C are both ATPases involved with the HSP70 family and Hsp90 complex, respectively. The most unique (though shortest) list belongs to single parameter M-BISON. Nearly all of these true positives became significant due to DNA motif knowledge. As before, many of these unique genes make biological sense: YAL005C and YMR186W are both members of a heat shock protein family, YOR020C inhibits the activity of a heat shock protein, and YDR184C is involved in stress response. Interestingly, the top two genes of this list (YOR020C and YFL039C) share two motifs, Reb1 and ARS, neither of which have a known relation to heat or stress response. Each list has at least a few genes that do not appear in the other two, so all three methods of analysis have some value in identifying true positive genes.

We took a closer look at the behavior of M-BISON with respect to two gene stories: YOR020C and YBR045C. We displayed the effects knowledge had on these genes in the context of their knowledge neighbors and saw that the boosting or lowering of these genes was biologically justified. Those few genes whose behavior seemed contradictory to the majority of their network neighbors were explained by expanding the amount of the knowledge neighborhood considered. In addition, viewing M-BISON's effect on the Ndt80 transcription factor (YHR124W) in the context of its associated knowledge has suggested it to us as a potentially novel player in heat shock/stress response pathways. While the assumptions of the hypergeometric p-value in testing motif enrichment are not fully met (genes connected by knowledge will not have independent M-BISON scores), the overwhelmingly small p-value achieved by Ndt80 lends some confirmation to our hypothesis.

Using physical DNA binding data as a substitute for a DE gene gold standard, while biologically reasonable, is clearly not perfect. To investigate possible shortcomings with this approach, we looked in detail at the top 50 high-scoring genes from the lists generated by the B statistic with microarray data only, single parameter M-BISON, and empirical M-BISON (Figure 7 shows results for the first two methods). For each gene, we searched for background information implicating it in stress or heat response. Four of six such genes uncovered by single parameter M-BISON were not considered truly DE by the Hsf1p physical binding data, while the B statistic found only one such gene. Thus, a perfect gold standard might be expected to reveal even better M-BISON performance, at least in the highest-scoring part of the gene list.

It is worth considering what would constitute a perfect gold standard for validating a DE gene predictor. Ideally, a more accurate quantification of RNA (i.e. qRT-PCR) would be used, but obtaining such a measurement for every gene present on a microarray for a given condition is not feasible. Approaches used in the past include evaluating gene lists based on gene annotations or prior knowledge of a condition, but these methods are at best indirect and apply to only a subset of all genes. We believe that using a related high-throughput assay like ChIP-chip for a silver standard is a good compromise, as TFs are expected to regulate the expression of many of their target genes. By choosing a closely related knowledge source like genome-wide motifs, which should predispose DE genes to be those containing motifs used by TFs, we hope to further minimize discrepancy between the silver standard and the unknown truth. We note that though a standard like this will always miss some true positives (i.e. those resulting from secondary targets), it will still be useful in comparing relative performances between DE predicting methods.

We compared M-BISON performance to that of a competing method, GeneRank, using the heat shock dataset. We note that GeneRank depends on a single parameter, *d*, whereas the M-BISON model requires specification of three: *α*^(*NDE*)^, *α*^(*DE*)^, and *α*^(*b*)^, the first two of which are non-trivial to estimate. In terms of AUC and pAUC^.2^, M-BISON outperforms GeneRank, both in performance using optimal parameter choices and, more importantly, in performance when knowledge of the truth (and thus the values of the optimal parameters) is withheld. We believe the reason for such improved performance is two-fold. First, because M-BISON utilizes a probabilistic framework, uncertainty present in gene expression data is automatically incorporated into the model. This is in contrast to GeneRank, which uses log ratios of expression. We would expect this to improve results in much the same way that a statistically sound method of detecting DE genes with microarray data improves over fold-change based methods. We also note that though M-BISON requires a Gibbs sampling procedure for inference, the computational time required to evaluate a parameter combination is approximately the same as is needed by GeneRank, which must solve a large system of linear equations. Second, when considering the good performance of empirical M-BISON, we note that this approach effectively tries many combinations of parameter values for each gene, choosing the combination that yields the most significant p-value. In contrast, GeneRank uses one value of the parameter *d*, and thus we would expect better M-BISON performance in cases where different DE genes would benefit from different strengths of knowledge influence (i.e., differing values of *α*^(*NDE*) ^and *α*^(*DE*)^).

One additional advantage of using a probabilistic model in M-BISON is that any genome-wide experimental modality using scores that can be converted to probabilities is a candidate for M-BISON. Thus, ChIP-chip, protein microarray, and even genome-wide association data might benefit from such a knowledge integration approach.

Several challenges remain in using M-BISON for its intended purpose. As mentioned above, one difficulty is choosing optimal parameter values in data-knowledge configurations that do not benefit from the use of the empirical approach. A second and related challenge is the proper handling of knowledge that is uninformative to the condition queried by the microarray experiment. In this case use of any amount of knowledge would be expected to introduce error into the results. By treating all knowledge as a structured graph, we believe we can solve this issue in the process of solving the first challenge, by predicting the unknown configuration of data and biological knowledge. Lastly, we should accommodate more complicated forms of knowledge. One example of this is continuously valued (rather than binary) connections between genes. This would prove useful if Gene Ontology semantic similarity measures between genes were used as knowledge. Another example is use of multiple forms of knowledge, each separately parameterized. With more than a few sources, a grid search becomes computationally prohibitive, and thus a more sophisticated approach to sampling parameter values becomes necessary.

## Conclusion

In this work we have developed, validated, and made available M-BISON, a probabilistic method for integrating biological knowledge with gene expression data to identify DE genes. The method in its current state shows good performance in a useful subset of microarray data-biological knowledge configurations. In addition, our work provides a solid foundation to explore such a method's utility with other high-throughput data sources.

## Methods

### M-BISON

Our method allows relationships between genes, dictated by biological knowledge, to influence the prediction of DE genes from microarray data. Intuitively, it boosts the DE scores of genes that are connected through knowledge to many other high-scoring genes, and lowers the scores of genes that are connected to many low-scoring genes. This is in contrast to traditional methods of DE gene identification, which assign scores to each gene independently of all others.

As our method is concerned with identifying DE genes, we model average log ratios of expression between two conditions $\left\{{\hat{\beta }}_{k}\right\}$ and their residual sample variances $\left\{{\hat{\sigma }}_{k}^{2}\right\}$ as random variables in the same manner as Smyth [24]:

$$\begin{array}{c} {\hat{\beta }}_{k}|\beta_{k},\sigma_{k}^{2}\sim N(\beta_{k},\nu_{k}\sigma_{k}^{2})\\ {\hat{\sigma }}_{k}^{2}|\sigma_{k}^{2}\sim \frac{\sigma_{k}^{2}}{d_{k}}\chi_{d_{k}}^{2} \end{array}$$

where *v*_*k *_and *d*_*k *_represent the variance of ${\hat{\beta }}_{k}$ and residual degrees of freedom from estimating ${\hat{\beta }}_{k}$, respectively, and $\chi_{d_{k}}^{2}$ refers to a random variable having chi-square distribution with *d*_*k *_degrees of freedom. True values for the random variables {*β *_*k*_} and {*σ*_*k*_^2^} are unknown, and a non-zero value for *β *_*k *_implies that gene k is DE. Unlike Smyth, we do not assume that the estimators ${\hat{\beta }}_{k}$ and ${\hat{\sigma }}_{k}^{2}$ from different genes are unconditionally independent; instead, we model them as conditionally independent given the states of hidden variables {*h*_*k*_}:

{*h*_*k *_: *h*_*k *_= *I*(*β *_*k *_≠ 0), 1 ≤ *k *≤ *N*}

where *I*(·)is the indicator function and N is the number of genes on the microarray. Smyth derives a moderated t-statistic we will call *μ*_*k*_, which is a function of ${\hat{\beta }}_{k}$ and has the following conditional distributions [24]:

$$\begin{array}{c} \mu_{k}|h_{k}=0\sim t_{d_{o}+d_{k}}\\ \mu_{k}|h_{k}=1\sim {\left(1+\nu_{0}/\nu_{k}\right)}^{1/2}t_{d_{o}+d_{k}} \end{array}$$

where *d*_0 _and *v*_0 _represent empirical estimates of prior degrees of freedom and prior variance, respectively, and *t*_*d *_refers to a random variable having student's t-distribution with *d *degrees of freedom. In our model, the *μ*_*k *_| *h*_*k *_from different genes are also independent. These distributions lead directly to the two conditional densities of *μ*_*k*_, namely:

*p*(*μ*_*k *_| *h*_*k *_= 0)

*p*(*μ*_*k *_| *h*_*k *_= 1)

We model expression dependencies between genes through the hidden variables {*h*_*k*_} with an undirected graphical model. Our approach modifies that of the classic Boltzmann machine (BM): a stochastic, binary state network with hidden and observed nodes and weights that operate on node pairs [29]. Versions of this model have been used in other areas of biology, such as protein and gene function prediction [30,31]. Pairwise connections in the graph indicate a functional relationship between genes given by the knowledge. Inference is achieved through Gibbs sampling, where the hidden state of node *k *during a given iteration is set to 1 with probability *p*_*k *_(0 otherwise):

$$p_{k}=\frac{1}{1+\exp\left\{-\Delta E_{k}\right\}}$$

with

$$\Delta E_{k}=\sum_{i↔k,i\neq k}\left[\alpha^{\left(DE\right)}s_{i}-\alpha^{\left(NDE\right)}(1-s_{i})\right]-\alpha^{\left(b\right)}+\ln\left[\frac{p(\mu_{k}|h_{k}=1)}{p(\mu_{k}|h_{k}=0)}\right]$$

Here, *i *↔ k denotes an edge between genes *i *and *k *and *s*_*i*_∈ {0,1} is the state of the hidden node *h*_*i *_sampled in the course of running the model. The model has three unknown parameters: *α*^(*NDE*)^, *α*^(*DE*)^, and *α*^(*b*)^. The first two are specific to the knowledge source; they determine the extent to which the knowledge can decrease or increase the probability of DE, respectively. The third parameter is a shared bias term which can be thought of as a prior on the number of genes with *h*_*k *_= 1. In our experiments, we have found that changing this parameter has no effect on overall performance, although the precise values of *α*^(*NDE*) ^and *α*^(*DE*) ^that lead to optimal performance may be slightly different. Thus, we fix *α*^(*b*) ^at 2*ln [(1 - Pr(*h*_*k *_= 1)/Pr(*h*_*k *_= 1)], with Pr(*h*_*k *_= 1) user-defined, reducing the parameter space and effectively enforcing a prior probability of differential expression. The model possesses the following density function:

$$\begin{array}{l} p(h=s,\mu |\alpha )=\frac{1}{Z(\alpha )}\exp\left\{\sum_{i<j}I(i↔j)\left[\alpha^{\left(NDE\right)}I(s_{i}=s_{j}=0)+\alpha^{\left(DE\right)}I(s_{i}=s_{j}=1)\right]-\sum_{i}\alpha^{\left(b\right)}s_{i}\right\}\\ \times \prod_{i}p(\mu_{i}|h_{i}=s_{i}) \end{array}$$

with **s **= [*s*_1_,...,*s*_N_]^T ^and *Z *a normalization constant over all possible state configurations.

**Figure 11:**
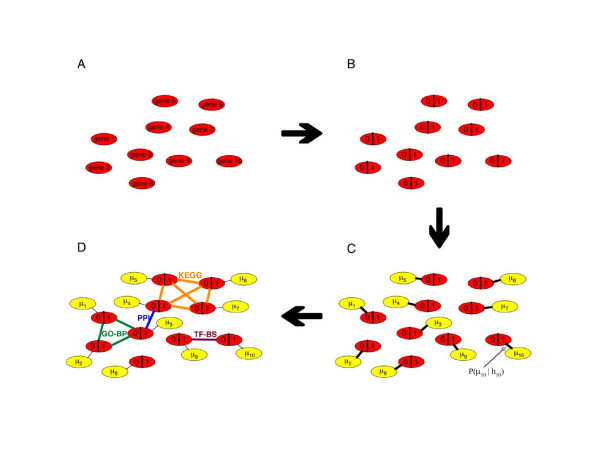
**Graphical depiction of M-BISON model**. Each gene is represented by a red node (A), which takes on hidden binary values for differential expression (*h*_*k*_'s) (B). Microarray data (yellow) are incorporated probabilistically (*μ*_*k*_'s) (C), and biological knowledge is used to connect genes that share experimental or annotation information (green, purple, blue, orange edges) (D).

One iteration of Gibbs sampling involves estimating the state of each of the N genes in a random order. Full inference using the model is achieved by running Gibbs sampling for a number of burn-in iterations followed by a larger number of sampling iterations. During sampling, states of the hidden nodes are recorded at every iteration. Gene *k *is assigned a DE score in the following manner:

$$B_{k}^{*}(\alpha )=\ln\left[\frac{\langle h_{k}\rangle }{1-\langle h_{k}\rangle }\right]$$

with <·> denoting the sample mean of the states held by *h*_*k *_in the course of sampling. The score is so labeled to emphasize that it is a knowledge-informed modification of the *B *score used by Lonnstedt and Speed [24] and that it is a function of the parameter values ***α***. In our experience, burn-in iterations on the order of 10% of N and sampling iterations on the order of N are sufficient for adequate convergence and resolution, respectively. On a 2.4 GHz AMD Opteron, full inference with one set of parameter values on a 6000 gene dataset takes approximately 30 s. We assessed convergence by running the algorithm several times on the same data using different random number generator seeds and comparing the results. Using the aforementioned number of burn-in and sampling iterations, the resulting DE scores were always qualitatively the same. A graphical depiction of the M-BISON model is shown in Figure 11.

### Standard parameter estimation methods

M-BISON requires careful choice of values for *α*^(*NDE*) ^and *α*^(*DE*) ^to yield good performance, and these optimal values are *a priori *unknown. To learn these values in an unsupervised manner, we first considered using the standard Boltzmann machine learning algorithm [29]. This approach applies gradient descent to maximize the likelihood of the observed data given the parameters. Due to the large number of possible states in the model, Gibbs sampling is used to generate samples from the distribution over states, and these samples are used to calculate the gradient. Unfortunately, this learning algorithm is known to be extremely slow for models with large numbers of nodes (our yeast data set has ~6000) [32]. Approximate methods have been devised to resolve these difficulties, but as our model has only two free parameters, we tried a grid search to identify parameter values that maximize data likelihood. Using a cross-validation strategy to avoid overfitting, we discovered that for real datasets, maximum likelihood parameter estimates were not those that yielded the best performance. Faced with this discrepancy, we turned to empirical approaches instead of continuing to explore more elaborate likelihood-based ones.

### Empirical method to combine results from multiple runs

The parameterization of M-BISON trades off complexity in model specification for a small number of free parameters. We expect instances where different DE genes would benefit from different strengths of knowledge influence, so we investigated an approach that scores each gene based on a version of the model run with a potentially unique set of parameter values. In essence, this method integrates results from runs using many different combinations of parameter values.

First, we choose initial parameter values $\alpha_{0}^{\left(NDE\right)}$ and $\alpha_{0}^{\left(DE\right)}$ and run M-BISON on the expression data and knowledge. This generates an initial vector of scores B*(***α***_0_). Next, we generate *M *randomly permuted expression datasets by shuffling the gene labels of the expression matrix. We run M-BISON on each one of these datasets along with the unmodified biological knowledge. This yields for each gene *k *a null distribution of scores $B_{k}^{*0}$ (***α***_0_). Using each null distribution we convert the original scores to p-values by computing for each gene *P*_k_* (***α***_0_):

$$P_{k}^{*}(\alpha_{0})=M^{-1}\sum_{i}I(B_{ki}^{*0}\geq B_{k}^{*})$$

This gives a new vector of scores P* (***α***_0_) = [*P*_1_* (***α***_0_) ⋯ *P*_N_* (***α***_0_)]^*T*^, which alone provides a way to eliminate false positive high scores that arise irrespective of the expression data.

We then repeat the above procedure across a *q *× *r *sized grid of varying parameter values given by all pairwise combinations of $\left\{\alpha_{i}^{\left(NDE\right)}\right\}$ : 0 ≤ *i *≤ *q *- 1} and $\left\{\alpha_{j}^{\left(DE\right)}\right\}$ : 0 ≤ *j *≤ *r *- 1} (not repeating the first parameter combination tested above). Overall, this equates to testing q * *r *parameter combinations {***α***_*t*_: 0 ≤ *t *≤ q * *r *- 1}, with $\alpha_{t}={\left[\alpha_{i}^{\left(NDE\right)}\alpha_{j}^{\left(DE\right)}\right]}^{T}$. From this procedure we obtain q * *r *vectors of scores {P* (***α***_*t*_)}, which we combine by taking the minimum p-value obtained for each gene over all runs:

$${\left[\underset{0\leq t\leq q*r-1}{\min}\left(P_{1}^{*}(\alpha_{t})\right)\underset{0\leq t\leq q*r-1}{\min}\left(P_{2}^{*}(\alpha_{t})\right)\cdots \underset{0\leq t\leq q*r-1}{\min}\left(P_{N}^{*}(\alpha_{t})\right)\right]}^{T}$$

We call this composite vector of scores $P_{\min}^{*}$. The above procedure allows the final score for each gene to come from the parameter value combination giving the most statistically significant result. In this way, we hope to take advantage of the strengths of all parameter value combinations in the grid without having to choose any single set. Pseudocode for the overall method is provided [see Additional file 2].

### Data simulation

We simulate microarray data as normally distributed random variables whose parameters are determined from a highly replicated yeast expression dataset [33]. DE genes are drawn from a *N*(*m*_*i*_, .2) distribution with *m*_*i *_~ *N*(0, .25); NDE genes are drawn from a *N*(0, .2) distribution. We achieve differing accuracies of array datasets by sampling different numbers of experimental replicates.

We simulate biological knowledge using a modified geometric random graph (GRG) model. Przulj et al. have shown that one example of a complex biological network, protein-protein interactions, are best modeled by GRGs [34]. We have found that a modification of this model is also quite amenable to customization in terms of several graph theory parameters: mean degree (number of connections) of all genes (*MD*), ratio of DE genes' mean degree to NDE genes mean degree (*β*), mean clustering coefficient (*MCC*; a measure of node connection density) [35], and relative connectivity (*RC*), defined as:

$$\begin{array}{ccc} \frac{p(i↔j|h_{i}=h_{j}=1,i\neq j)}{p(i↔j|h_{i}\neq h_{j})} & \text{with} & 1\leq i,j\leq N \end{array}$$

We simulate a knowledge source by first dropping N points randomly uniformly into a unit 3-dimensional cube. We add edges between any two points at Euclidean distance ≤ *r *from each other and remove all edges from nodes with degree ≤ *c*. The values for *r *and *c *are chosen empirically (and often separately for DE and NDE genes) to yield desired values of *MD*, *β*, *MCC*, and *RC*.

We evaluate M-BISON performance on simulated datasets given the known DE genes by calculating the area under the ROC curve (AUC) at a false positive rate cutoff of both 0.2 and 1. The R package *ROCR *[36] was used for this purpose.

### Biological validation

To demonstrate the utility of M-BISON on real biological data, we chose a yeast dataset querying the heat shock transcription factor gene (*hsf1*). We use gene expression data from [21] and biological knowledge from [22]. For the latter, we included any upstream conserved motifs at or above a score of 55, and we connect two genes in the knowledge graph if they share at least one motif. As a silver standard, we use ChIP-chip results from [23], which reports 165 unique ORFs bound by Hsf1p. Thus, we evaluate M-BISON's performance, in terms of AUC, of predicting likely heat shock-related DE genes (physical Hsf1p binding sites) given relevant expression data and biological knowledge. A table listing each gene's expression value, M-BISON score, and upstream motifs is provided [see Additional file 1].

### Comparison to existing methods

We downloaded the MATLAB code for GeneRank, available as an additional file on the BMC Bioinformatics website. GeneRank relies on one parameter, *d*, which can take on values from 0 to 1. We ran GeneRank using each value of *d *from 0 to 1 in 0.01 increments on the *hsf1 *data and knowledge listed above and compared its performance to M-BISON using AUC.

### Differential expression story

We generated graphical representations of all knowledge networks using the *tkplot *function of the R *igraph *package. Layouts were created using the fruchterman-reingold algorithm with default parameters.

Hypergeometric p-values for motif enrichment were calculated using the *phyper *function in the R *stats *package. This function computes the probability due to chance of seeing a given or better enrichment of motifs at the top of a ranked list.

## Availability and requirements

We implemented M-BISON and its associated evaluation functions as a documented R package, available at simtk.org under the project name "m-bison".

## Authors' contributions

BJD designed and implemented the algorithm, performed the experiments, and drafted the manuscript. RBA designed and coordinated the study and helped to draft the manuscript. All authors read and approved the final manuscript.

## Supplementary Material

Additional file 1 Listing of each gene, its original microarray score, M-BISON scores, and upstream motifs from the *hsf1 *dataset. Click here for file

Additional file 2 Pseudocode for the M-BISON method. Click here for file
