# Supplementary material for: M-BISON: Microarray-based integration of data sources using networks
Source: BMC Bioinformatics. 2008 Apr 25;9:214. doi: 10.1186/1471-2105-9-214 (PMC2396182; doi:10.1186/1471-2105-9-214)
Supplement: Additional file 2 — Pseudocode for the M-BISON method. [file 1471-2105-9-214-S2.rtf]

Method:  getScoresFromOneParameterCombination(dataConditionalDensities, knowledge, 
  parameterCombination, burnIter, sampIter)
Input: 	  dataConditionalDensities – conditional densities of microarray data probing condition of 
  interest calculated from limma package
  knowledge – adjacency matrix describing knowledge connectivity
  parameterCombination – pair of values, one each for a(NDE) and a(DE) 
  burnIter – number of burn-in iterations
  sampIter – number of sampling iterations
Output:   scores – vector of M-BISON DE scores, one for each gene

  1   states  initializeStatesAtRandom()   // Initialize states of network
  2   // Gibbs sampler
  3   // Burn-In
  4   for (burnIter + 1)
  5   	for (randomizeOrder(states))
  6	      // Calculate most likely state {0,1} given knowledge-neighboring states and parameter 
  7   	      // values (see Methods section of manuscript)
  8	      state  generateStateWithBoltzmannProbability(knowledge, parameterCombination, states, 
  9 		      dataConditionalDensities)
10   // Sampling
11   stateCounts  states   // Initialize state counts
12   for (sampIter – 1)
13   	for (randomizeOrder(states))
14 	       state  generateStateWithBoltzmannProbability(knowledge, parameterCombination, states, 
15		      dataConditionalDensities)
16   	stateCounts  stateCounts + states   // Increment state counts
17   // Calculate scores
18   scores  log(stateCounts/sampIter) – log(1 – stateCounts/sampIter)

Method:  getEmpiricalPValues(alphaNDEValues, alphaDEValues, nPerms, dataConditionalDensities, 
  knowledge, burnIter, sampIter)
Input: 	  alphaNDEValues – vector of values of a(NDE) used to calculate empirical p-values
	  alphaDEValues – vector of values of a(DE) used to calculate empirical p-values
	  nPerms – Number of randomly permuted datasets to use for p-value calculation
  dataConditionalDensities – conditional densities of microarray data probing condition of 
  interest calculated from limma package
  knowledge – adjacency matrix describing knowledge connectivity
  burnIter – number of burn-in iterations
  sampIter – number of sampling iterations
Output:   pvalues – vector of M-BISON empirical p-values, one for each gene

  1   allPVals  0   // Initialize matrix of p-value vectors for all parameter combinations
  2   // Iterate over grid of a(NDE) and a(DE) 
  3   for(alphaNDEValues)
  4   	for(alphaDEValues)
  5	      parameterCombination  concatenate(alphaNDEValue, alphaDEValue)
  6	      // Calculate scores for real data with current parameter combination
  7	      scores  getScoresFromOneParameterCombination(dataConditionalDensities, knowledge, 
  8		        parameterCombination, burnIter, sampIter)
  9	      singlePVals  0   // Initialize vector of p-values for current parameter combination
10   	      // Iterate over number of permuted datasets
11	      for(nPerms)
12		// Randomize data
13		nullConditionalDensities  randomizeOrder(dataConditionalDensities)
14		// Calculate null scores
15		nullScores  getScoresFromOneParameterCombination(nullConditionalDensities, 
16			         knowledge, parameterCombination, burnIter, sampIter)
17		// Increment p-value vector
18		singlePVals  singlePVals + elementWiseIsGreaterThanOrEqualTo(nullScores, scores)
19	      singlePVals  singlePVals / nPerms
20  	      // Keep track of current p-value vector
21	      allPVals  concatenate(allPVals, singlePVals)
22   // Calculate final vector of p-values by taking each gene's minimum p-value
23   pvalues  geneWiseMinimum(allPVals)
